# Behavioural Psychology of Unique Family Firms Toward R&D Investment in the Digital Era: The Role of Ownership Discrepancy

**DOI:** 10.3389/fpsyg.2022.928447

**Published:** 2022-07-28

**Authors:** Muhammad Zulfiqar, Weidong Huo, Shifei Wu, Shihua Chen, Ehsan Elahi, Muhammad Usman Yousaf

**Affiliations:** ^1^School of Finance and Trade, Liaoning University, Shenyang, China; ^2^International Business School, Dalian Minzu University, Dalian, China; ^3^School of Business Administration, Dongbei University of Finance and Economics, Dalian, China; ^4^School of Economics, Shandong University of Technology, Zibo, China; ^5^Lyallpur Business School, Government College University, Faisalabad, Pakistan

**Keywords:** family firm behaviour, ownership discrepancy, voting rights, cash-flow rights, R&D investment

## Abstract

This study examines the R&D investment behaviour of different types of family-controlled firms with the moderating role of ownership discrepancy between cash-flow rights and excess voting rights by using the sufficiency conditions’ theoretical framework of ability and willingness developed by De Massis. It uses data from family firms that have issued A-shares from 2008 to 2018. They used pooled OLS regression for data analysis and Tobit regression for robustness checks. This study classifies family firm types into two categories, namely, the lone-controller family firms (LCFFs) and the multi-controller family firms (MCFFs), with each being further classified as “excess” or “no excess” voting rights. Both LCFFs without excess voting rights and MCFFs with excess voting rights have the “ability” and “willingness” toward R&D investment. LCFFs with excess voting rights and MCFFs without excess voting rights only have the ability but low willingness to invest in R&D. The study also establishes that Chinese family-controlled firms are heterogeneous toward risky investment. To the best of our knowledge, this study is the first to differentiate Chinese family firms by their unique ownership structure characteristics in investigating the effect of the family firm structure on R&D investment. The study is a novel attempt to test the willingness and ability framework of LCFFs and MCFFs. Previous studies based on agency theory have tacitly assumed that ability and willingness exist in family-controlled firms. However, this study challenges this implicit assumption.

## Introduction

Family firms are important to researchers in related fields. Family firms face several challenges, such as innovation goals, agency problems, market competition, governance improvement, intergenerational transmission, and international competition. According to behavioural agency theory, family firms’ controllers are loss-averse regarding their socio-emotional wealth and might ignore some business opportunities (Chrisman and Patel, 2012). Family firms are defined and designed by family firms’ controllers and their other major family shareholders, having several aims and visions, such as how the family firms potentially benefit their families across generations (Chua et al., 1999; Bennedsen et al., 2010; Patel and Chrisman, 2014). Family-controlled firms are less willing to invest in research and development (R&D) than non-family-controlled businesses (Chen and Hsu, 2009; Munari et al., 2010; Block, 2012; Chrisman and Patel, 2012). However, family firms are more oriented toward long-term orientation than non-family firms (Cassia et al., 2012).

Innovation in family-controlled businesses has attracted substantial attention from scholars in recent years (Magistretti et al., 2019). Recently, meta-analyses by Duran (2016) established that the lone-founder family firms are positively significant with the innovation input containing (R&D) investment. Surprisingly, different scholars found a substantial dissimilarity among lone-founder firms and family firms (Miller et al. 2010, 2011; Block, 2012). Lone-founder family firms have an insignificant effect on R&D investment (Miller et al. 2010, 2011; Block, 2012). Additionally, Block (2012) found that lone-founder family firms are more interested to invest in R&D than family and non-family firms. These findings suggest that remarkable differences exist in R&D investment behaviour among different types of family firms. If we review the literature then we find that the majority of the research has been conducted on the comparison of R&D investment behaviour of family firms and non-family firms, whether we talk about risk preference (Fang et al., 2021), gender diversity on board (Hernández-Lara and Gonzales-Bustos, 2020), internationalisation (Lin and Wang, 2021), board chairs and R&D investment (Jiang et al., 2020), and many more. Scarce research is found in the literature that deals with the R&D investment behaviour of different types of family firms only. Therefore, gaps exist regarding R&D investment behaviour within different types of family firms. These findings also indicate that family firms are naturally heterogeneous. Within the control mechanism, how kinfolks with the controlling rights affect decisions on R&D of family firms remains lacking in family business research.

According to Jin and Park (2015), a firm’s performance can be affected by the separation of controlling rights and cash-flow rights. Jin and Park (2015) confronted the issue of controlling minority shareholding, which, according to Faccio and Lang (2002), is found around the world. In general, it affects the firm’s performance negatively because controlling minority shareholders have more voting rights with less cash-flow rights and result in a separation of voting rights and cash-flow rights. This issue creates agency issues in the firms. Contrary to these views, Jin and Park (2015) concluded that the separation of cash-flow and voting rights has a significant positive effect on the performance of the firms and moderates the R&D expenditure by following agency theory. Several previous studies have established that in East Asian countries such as Korea, China, and Taiwan, the controlling shareholders’ ownership concentration is extremely high (La Porta et al., 1999; Claessens and Fan, 2002; Jiang and Kim, 2015; Kim and An, 2018). In Western countries, the typical agency problem arises from the conflicts between management and shareholders. In East Asian countries, many agency issues arise because of majority and minority shareholders. Therefore, majority shareholders attempt to keep more controlling rights than cash-flow rights (Lemmon and Lins, 2003; Cornett et al., 2008; Kim and An, 2018).

Aiello et al. (2020) used firm-level data and concluded that R&D investments have a positive impact on firm productivity in a sample of European manufacturing firms over the period 2007–2009. The researchers further found that R&D investment was more profound in family firms as compared to non-family firms. Eng et al. (2021) found that R&D investments in family firms are more dependent on prior R&D as compared to non-family firms. The researchers further found that R&D investments in family firms are motivated by internal as well as external cash-flows more than non-family firms. Wang et al. (2022) stated that the level of family involvement plays a notable role in R&D investment decisions in family firms. The researchers concluded that family involvement negatively affects the R&D intensity in Chinese family firms, while potential gains through R&D investment positively affect the socio-emotional wealth of the company in high-tech Chinese family firms. Above studies have mainly focused on the comparison between family and non-family firms, and limited research has focused on the R&D investment behaviour of different types of family firms. We need to classify the family firms because family firms are heterogeneous (Miller et al., 2011; Daspit et al., 2018; Fang et al., 2019). Many scholars have stated that the major source of heterogeneity in family firms is the nature of family involvement in governance, ownership, and management (Miller et al., 2011; Daspit et al., 2018; Fang et al., 2019). Anderson and Reeb (2003) stated that family firm heterogeneity depends on the combination of family and non-family members in the management.

The mixed results of studies on family firms such as Miller et al. (2010, 2011), and Block (2012) which only focused on R&D investment, are inadequate to understand the competitive behaviour of family-controlled firms. Therefore, for the first time, we differentiate the family firms on the basis of the unique ownership structure characteristics, as described by Bozec and Di Vito (2019). Moreover, we investigate the behaviour of a different type of family-controlled firm regarding R&D investment with and without excess voting rights. The presence of excess voting rights means that the family firm’s controllers have more controlling/voting rights than cash-flow rights. The absence of excess voting rights means that the family firm’s controllers have controlling/voting rights equal to cash-flow rights.

There are three forms of family-controlled firms. In this study, we explore two forms and use the third for reference. The first type is the lone-controller family firm (LCFFs), which is solely a single-person entrepreneur firm. In the LCFF, the organiser or controller is an individual. No family kin exists in the parent firm or the controlling shareholder firm owning shares and administering supervision. Many studies use this approach for considering family firms (Miller et al. 2010, 2011; Block, 2012). The second type of family-controlled firm is the multi-controller family firm (MCFFs). Apart from the actual controller, at least one family member with kinship holds, manages, and controls the family business of the listed company or the supervisory shareholder company. LCFF firms are also referred to as a non-board family (NBF), in which no other family member, except the single controller, plays any controlling role on the board (Martínez and Requejo, 2017). In our dataset, nearly 11% of the firms have changed their family firm status from LCFFs to MCFFs.

Therefore, we attempt to explore the family firms’ types and R&D relationships by adopting the adequate circumstances outlined to recognise the ability and willingness paradox (De Massis et al., 2014; Bozec and Di Vito, 2018). In other words, the *ability* is the allocation, disposing of, and adding of the firm’s resources or assets at the discretion of the family firm controller. By contrast, we define *willingness* as the attitude or behaviour of a family firm controller’s personality (disposition) based on the intentions, goals, and motivations toward the progress of the firm (De Massis et al., 2014; Bozec and Di Vito, 2018).

This study contributes to knowledge in several ways. First, the study fixes the spotlight on different types of family businesses, i.e., LCFFs and MCFFs. By highlighting the different kinds of family-controlled firms, the study checks the instrumental key roles of excess voting rights in R&D investment behaviour. By following the De Massis et al. (2014) framework, we propose the significance of having the two essential conditions (ability and willingness) to describe a pattern and design of behaviour. Our study concerns the investment attitude and behaviour of lone- and multi-controller family firms regarding R&D investment. Previous studies (Miller et al., 2011; Block, 2012) based on agency theory have tacitly assumed that ability and willingness exist in family-controlled firms. However, we challenge this implicit assumption. The founder-controlled family firms, which have ability and willingness, invest more in R&D than do other types of firms (Bozec and Di Vito, 2018). Second, this study also examines the critical effect of more control rights of LCFFs on the ability and willingness to invest in R&D. This study then compares the findings with multi-controller family firms. Third, unlike previous studies that did not differentiate family firms, this study inspects the behaviour of different types of family firms with high and equal voting/controlling rights and cash-flow/ownership rights.

## Literature and Hypothesis

### Family-Controlled Firms Without Excess Voting Rights

#### Long-Term Risky Decision

According to Miller and Sardais (2011), voting rights empower a shareholder to cast their votes on important corporate issues such as the board of director selection and strategic decision-making of a company, which require approval from shareholders (Wang et al., 2020). In contrast, cash-flow rights enable a shareholder to receive the shares from a company’s profit. Common shares issued by a firm have voting rights as well as cash-flow rights, while preferred shares only have cash-flow rights (Scherrer and Fernandes, 2021). This shows the importance of voting rights over cash-flow rights. Family firms without excess voting rights refer to businesses in which the controllers have voting rights not exceeding cash-flow rights. Shi et al. (2022) argued that excess voting rights are more useful for family firms as compared to non-family firms in order to prevent financial misconduct. Jara et al. (2021) also argued that excess voting rights of family members may not lead toward poor governance in family firms.

Cash-flow rights of group-controlling stockholders are one of the most significant determining factors of R&D intensity (Sung et al., 2017). The outcomes of R&D investments frequently need considerable time, and thus, patience is needed. The family firms typically want to hold control for a long time, especially in LCFFs, where the individual controller enjoys the rights for a prolonged period. Family owners are more committed to their employees and organisation because they remain in the firm for a longer period than non-family owners; therefore, they can induce more effective R&D investment compared with their counterparts (Chen et al., 2013).

#### Ownership Concentration Is a Powerful Tool

Ownership concentration provides power to the family firm for healthy decision-making (ability). Moreover, OC provides a powerful incentive instrument for the maximisation of the firm’s value (Jensen and Meckling, 1976; Shleifer and Vishny, 1997). Apart from agency theory, family firms, by the substantial cash-flow rights, are willing to offer incentives (i.e., willingness) in R&D engagements (activities) (Bozec and Di Vito, 2018). This finding is explained by the fact that the family firm’s controller, as a substantial shareholder, ultimately gains the benefits of the increasing value of the firm. Therefore, the ownership concentration indicates that a majority of the substantial shareholders (i.e., the family firm’s controllers) are also willing to invest their wealth (Chen and Hsu, 2009). Personal wealth is closely linked to the firm’s wealth, thereby inspiring the controllers’ willingness to take risks (Duran, 2016).

Some firms may show risk-aversion behaviour due to certain conditions such as founding region, availability of financial resources, and many other notable conditions (Hu and Hughes, 2020; Madanoglu et al., 2020). According to Schulze et al. (2002), high ownership concentration is also associated with risk-aversion behaviour. But high ownership concentration also offers decision power (ability) and incentives (willingness) based on the broader prospect. According to Zulfiqar and Hussain (2020), shareholders can exercise their statutory rights in the strategic decision-making of a company due to ownership structure concentration. Based on the positive attitude toward risk-taking investment (Schmid et al., 2014), we predict that LCFFs in the non-existence of excess voting rights are keener (willingness) to invest in R&D expenditure than MCFFs and non-family firms.

#### Non-economic Objective

Although family and non-family firms must pursue economic goals, many family firms also have non-economic goals. Family businesses may wish to give benefits to their families specifically for non-financial purposes, take long-term risk-oriented decisions for the next generation, and create what Gómez-Mejía et al. (2007) termed socio-emotional wealth. The LCFFs and MCFFs may also have similar objectives, including creating jobs for family members, enhancing security, and maintaining corporate control. Some studies such as Chrisman and Patel (2012) used the behavioural agency model and suggested that family-controlled firms have a myopic loss aversion to their socio-emotional wealth and show less interest in investing in risky long-term projects such as R&D investment.

The LCFFs and MCFFs both have the ability (discretionary power) to utilise and dispose of resources and diverge in terms of willingness to participate in R&D investment. First, we expect that the LCFFs without excess voting rights have both the ability and willingness sufficiency conditions in R&D investment than the other firms by linking the above discussion. Second, the attitude and behaviour of family-controlled firms are narrowed by their loss aversions regarding their socio-emotional wealth. Family-controlled firms may take family-concerned decisions and participate in non-economic activities.

When managers’ interest in control retention decreases due to higher cash-flow rights of controlling shareholders, then firms are likely to engage in higher R&D activities (Sung et al., 2017). Sung et al. (2017) concluded that a negative relationship exists between cash-flow rights of the controlling shareholders and agency cost, and a positive relationship exists between cash-flow rights of controlling shareholders and R&D intensity. The author also found that R&D intensity is higher for group-affiliated firms when either the difference between cash-flow rights and voting rights is lower or the cash-flow rights of group-controlling shareholders are higher.

Moreover, these behaviours of LCFFs and MCFFs are linked with the behavioural agency model (BAM). According to Gomez–Mejia et al. (2014), family firms’ risk-taking behaviour is well understood by this model. While linking the behavioural agency model with family firms, the major focus of the firm is socio-emotional wealth (Gomez–Mejia et al., 2014). This model also suggests that loss-averse family firms that attempt to preserve their socio-emotional wealth tend to invest less in R&D investment (Chrisman et al., 2012). According to Gomez-Mejia et al. (2011), this model has been used explicitly to explain why a family’s desire to preserve the socio-emotional wealth associated with firm control can result in executive entrenchment. Keeping in view the reviewed literature and behavioural agency model, we thus predict that the MCFFs without excess voting rights have less willingness to invest in R&D than LCFFs.

Lone-controller family firms (LCFFs) without excess voting rights are positively associated with R&D investment.

Multi-controller family firms (MCFFs) without excess voting rights are negatively associated with R&D investment.

### Family-Controlled Firms With Excess Voting Rights

In this scenario, controller shareholders have voting/controlling rights more than cash-flow rights. Excess voting rights are well-known worldwide, especially in family-controlled businesses (La Porta et al., 1999; Faccio and Lang, 2002). This privilege is often achieved through the practice of dual-class share or pyramidal ownership structure. In the dual-class share structure, firms are issued two classes (A and B) of common shares. For example, an A-class share is equal to one vote per share, and a B-class share could be equivalent to ten votes per share. Typically, family firms buy the B-class shares because they seek to gain additional control over the firms. Dual-class shares permit companies to access equity financing from the capital market without losing their control of the companies. This type of practice leads to agency conflicts when the firms have more control rights than cash-flow rights (La Porta et al., 1999). When the firms have secured control rights even with less cash-flow rights, they impose their decision. Such a practice is a major source of agency conflicts. Several empirical studies prove that more voting or controlling rights than ownership/cash-flow rights decrease a firm’s value (Villalonga and Amit, 2006), apart from its stock and accounting returns (Joh, 2003; Baek et al., 2004).

Excess voting rights violate the one-share-one-vote principle (Harris and Raviv, 1988). According to Burkart and Lee (2008), the unfair distribution of power due to excess voting rights disturbs the incentive structure of shareholders. Owing to this unfair distribution of power, this violation is undesirable and can negatively affect the firm value and social welfare of the firm (Harris and Raviv, 1988).

Controlling shareholders normally externalise the costs related to investment decisions when cash-flow rights are smaller (Bebchuk et al., 2000). The gap between voting rights and cash-flow rights is directly related to controlling shareholder incentives due to suboptimal investment decisions. Bebchuk et al. (2000) also stated that the gap between cash-flow rights and voting rights should have a negative relationship with R&D activities irrespective of the expropriation resulting from either suboptimal investment decisions or tunnelling procedures. Excess voting rights empower an owner to extract extra benefits from the firm by lowering the cost of managing the firm with lower financial engagements. However, this extra benefit of controlling shareholder is obtained at the cost of other shareholders (Miller and Le Breton-Miller, 2006; Villalonga and Amit, 2006).

We propose that the excess voting rights are essential determinants to change (LCFF’s willingness in R&D investments). Family firms have full discretionary power (ability) to invest in R&D investments. Nevertheless, we opine that with excess voting rights, both types of family-controlled firms may have lesser incentives (willingness) to invest in R&D. BAM also suggests that family firms are risk-averse and have less willingness to invest in long-term risky projects (Chrisman et al., 2012).

Families with a disproportionate number of voting rights have more motivation and authority to govern their businesses in their own interests. Such discretionary rights allow for hefty remuneration packages for both the controllers and their family members in family enterprises. The controllers may not focus on costly investment undertakings. Owing to these activities, the family firms have limited available resources for R&D projects (Bebchuk et al., 2000). Ghafoor et al. (2021) concluded that CEOs in family firms are less motivated for R&D investments in the absence of excess voting rights, while family firms having CEOs with actual control rights show more inclined behaviour toward R&D investment.

We, therefore, argue that excess voting rights change the willingness conditions of LCFFs and encourage MCFFs to impede investment in R&D. In this situation, excess voting rights have an additional effect on the sufficiency condition (willingness) of lone family-controlled firms. We expect that LCFFs, with excess voting rights, have negative behaviour toward R&D investment. LCFFs, with their self-opportunistic actions, somehow ignore the minority shareholders’ rights. They do not work for the economic welfare of the other members. As discussed earlier, the reward of R&D investment takes a long time and requires patience, but perhaps the existence of excess voting rights of family firms adversely affects the long-term decision-making horizon. We argue that LCFFs have fewer incentives (willingness) to R&D investment with the existence of excess voting rights, and MCFFs would only aggravate that lower willingness. In this situation, family firms with excess voting rights take family-oriented opportunistic decisions; the cost of this type of investment behaviour should be externalised to minority shareholders (Bozec and Di Vito, 2018). Keeping in view all the above discussions, reviewed literature, and BAM, we consequently propose that the existence of excess voting rights would exacerbate the lesser willingness of family firms to engage in R&D investments. These above pieces of evidence and debates lead to the following two hypotheses:

***Hypothesis 1:*** *Lone-controller family firms (LCFFs) with excess voting rights are negatively associated with R&D investment.*

***Hypothesis 2:*** *Excess voting rights weaken the negative relationship between multi-controller family firms (MCFFs) and R&D investment.*

## Methodology

### Sample and Data

This study used data from family firms that have issued A-shares and are listed on the Shenzhen Stock Exchange or Shanghai Stock Exchange. The data were collected from the China Stock Market and Accounting Research Database (CSMAR). According to Carney et al. (2019), CSMAR is regarded as one of the leading database sources for publicly listed Chinese firms. Specifically for non-state-owned (non-SOEs) enterprises, CSMAR is a specialised database (Xu et al., 2015). We excluded all SOE firms from our data set. Data from 2008 to 2018 were collected for analysis. Firms with missing values of revenues, total assets, or total liabilities were removed from our samples. Equally, firms that had negative or zero values of total revenues, total assets, or common equity were eliminated. To eliminate the outliers from our sample, we kept our variables at the 1st and 99th percentiles. This same technique was followed by Kale and Shahrur (2007) and Carney et al. (2019). The final analysis yielded a final sample of 1,943 firms and 3,731 firm-year observations.

#### Control Proportion or Voting Rights

The actual controller has the proportion of controlling the listed company (%), also known as voting rights. The values of controlling rights have been directly taken from CSMAR. The CSMAR database reflects that the calculation of the controlling proportion of the family-controlled firms was based on calculation methods (Claessens et al., 1999, 2000; La Porta et al., 1999). Aslan and Kumar (2012) also calculated voting rights by following (Claessens et al., 1999, 2000; La Porta et al., 1999; refer to Appendix-C). The indicator is analysed from the perspective of the family as a whole, i.e., the proportion of control rights of listed companies owned by all actual controllers in the family members. When the actual controller is multi-person, the calculation is combined.

#### Ownership Proportion or Cash-Flow Rights

The actual controller has a percentage of ownership of the listed company (%) known as cash-flow rights. It refers to the ownership of a scheduled company owned by the actual controller through concerted action, multiple holdings, and cross-shareholdings. The values of cash-flow rights have been directly taken from CSMAR. The CSMAR database reflects the calculation of the cash-flow proportion of the family-controlled firms based on calculation methods (Claessens et al., 1999, 2000; La Porta et al., 1999). Aslan and Kumar (2012) also calculated voting rights by following (Claessens et al., 1999, 2000; La Porta et al., 1999; refer to Appendix-C). The indicator is analysed from the perspective of the family as a whole and the proportion of ownership of listed companies owned by all actual controllers who are family members.

#### Separation Proportion Between Voting Rights and Cash-Flow Rights

The actual controller has the ownership ratio/control ratio. This indicator is analysed from the family perspective. When the family firms have voting rights higher than cash-flow/ownership rights, the firm is supposed to possess excess voting rights (Bozec and Di Vito, 2018).

#### R&D Investment

We used R&D investment as a dependent variable, which was measured by annual R&D expenditure divided by total assets at the end of the year. This measure of R&D intensity was used by several previous studies, such as those of Block (2012) and Bozec and Di Vito (2018).

This study generated the following two interaction terms, namely, LCFFs × with excess voting rights (H1) and MCFFs × with excess voting rights (H2). For regression output, this study used moderating variables as binary (dummy) (presented in Table 1). When the firms have excess voting rights, the variable is equal to 1; otherwise, it is 0. Subsequently, we used two variables, such as LCFF × with excess voting rights (H1) and MCFF × with excess voting rights (H2). We included binary (dichotomous) variables that categorise the LCFFs, MCFFs, and the other types of family firms included for reference.

**TABLE 1:** Regression results with binary (dummy) moderating variables.

	M3	M4
		
Variables	R&D	R&D
Lone controller family firms (LCFFs)	0.00342	
	(0.00512)	
Multi-controller family firms (MCFFs)		−0.00246
		(0.00433)
With excess voting rights	0.00159	−0.00895**
	(0.00251)	(0.00435)
LCFFss × With excess voting rights	−0.01110**	
	(0.00543)	
MCFFs × With excess voting rights		0.01120**
		(0.00477)
Leverage	−0.03040***	−0.03080***
	(0.00875)	(0.00865)
NOB_meetings	0.00046	0.00045
	(0.00032)	(0.00032)
CEO_tenure	0.00022	0.00023
	(0.00032)	(0.00032)
CEO overconfident	0.00347	0.00358
	(0.00243)	(0.00247)
CEO_duality	−0.00197	−0.00200
	(0.00206)	(0.00205)
Ind_director ratio	0.02630	0.02760
	(0.01840)	(0.01850)
Size	0.00390**	0.00394**
	(0.00198)	(0.00197)
Firm age	−0.00025	−0.00025
	(0.00029)	(0.00029)
ROE	−0.02150**	−0.02180**
	(0.00888)	(0.00891)
Board size	−0.00223*	−0.00217*
	(0.00114)	(0.00114)
Institutional	−0.00009	−0.00008
	(0.00021)	(0.00021)
Is_chairman_family	−0.00699**	−0.00712**
	(0.00304)	(0.00305)
Patent application	0.00001	0.00001
	(0.00001)	(0.00001)
Family CEOs	−0.00660***	−0.00702***
	(0.00191)	(0.00194)
Ultimate ownership	−0.00014	−0.00015
	(0.00011)	(0.00011)
Constant	−0.04480	−0.04340
	(0.03180)	(0.03220)
Year	Yes	Yes
Industry	Yes	Yes
R-squared	0.130	0.132

Robust standard errors are in parenthesis. ***, **, and * indicate P < 1, 5, and 10%.

To control the firm-specific characteristics, we utilised a set of control variables; the latter is acknowledged in the literature to influence R&D investment intensity (Czarnitzki et al., 2009; Block, 2012; Bozec and Di Vito, 2018). Accordingly, we used leverage as the firm’s total debt divided by total assets. In previous studies, CEO characteristics had effects on R&D investment. Thus, we included the tenure of the CEOs (CEO_Tenure) in our control variables. Numerous governance variables also include empirically tested governance variables that might also affect R&D investment intensity. We used governance variables such as CEO_Duality if the CEO is also the chairman of the firm; independent director ratio (Ind_Dir_Ratio) measured as the board size scaled by the number of independent directors; and total board size (Board_Size), which also represents the governance. We also included the number of board meetings (NOB_Meetings) in a given year as a control variable representing governance. Whether the chairman of the firm is a family member or not (Is_Chairman_Family) is used as a control variable (if the chairman is a family member, a value of 1 is assigned; otherwise, the value is 0). We used the firm size (SIZE) measured by the log of assets as a control variable. In a similar situation, firm size has been used by many scholars (Bozec and Di Vito, 2019). Return on equity (ROE) was also used to control the firm-specific characteristics, measured as net earnings scaled by total equity. In the control variables, we additionally included the age of the firm (Firm_Age). To control the effect of the institutional investor’s ownership on corporate investment, we included the proportion of voting rights held by institutions (Institutional) in our analysis (Miller et al., 2011; Bozec and Di Vito, 2018).

We took a 1-year lag on all independent variables and control for the year and industry-fixed effects. These steps decreased the potential biases in our empirical model from omitted variables and endogeneity. The family firm types and moderator variables are binary (dichotomous) and are usually fixed over time; thus, we performed our analysis using a pooled regression model (Wooldridge, 2003). The pooled regression model has been used by many scholars in similar situations (Schmid et al., 2014; Bozec and Di Vito, 2019; Fu, 2019).

### Empirical Model and Data


(1) $$R\&D_{i,t}=\alpha_{o}+\alpha_{1}familyfirmstypes\cdot_{i,t}+\alpha_{2}votingrights\cdot_{i,t}$$

+α3familyfirmstypes⋅i,tv*otingrights⋅i,t

$$+\alpha_{\text{j}}\,\sum C\,o\,n\,t\,r\,o\,l\,s_{i,t}+\varepsilon_{i,t}.$$

## Results

### Mean Comparison Analyses

“The term mean comparisons refers to the comparison of the average of one or more continuous variables over one or more categorical variables” (Salkind, 2010). This test helps researchers to evaluate whether means of two or more than two data set groups statistically differ from each other or not. In the current scenario, we are interested in comparing the LCFF and MCFF with and without excess voting rights, which makes this analysis appropriate for this research. We ran an analysis of the difference in means for three types of family firms, and Table 2 reports the t-statistics value. According to the t-statistics values, most of the variables were significant. In LCFFs and MCFFs, only the CEO tenure was insignificant. The number of observations was higher in the MCFFs than in the other two types of family-controlled firms. The average size of MCFFs was greater than that of LCFFs. The average value of R&D was lower in MCFFs than in the other two categories of family businesses. The mean value without excess voting rights was superior in MCFFs than in LCFFs. With excess voting rights, on average, was lower in MCFFs than in LCFFs as well as in other family firms.

**TABLE 2:** Mean comparison test.

Variables	Others	Multi-controller	T-test score	Others	Lone controller	T-test score	Others	Other family	T-test score
R&D	0.0147	0.0099	4.46***	0.0106	0.0142	−3.25***	0.0115	0.0169	−2.62***
With excess voting rights	0.664	0.463	20.86***	0.4634	0.6842	−22.45***	0.5390	0.4715	2.61***
Leverage	0.3947	0.3609	7.7***	0.3619	0.3947	−7.7***	0.3723	0.338	2.86***
NOB_Meetings	9.8429	9.4582	5.25***	9.4545	9.8838	−5.76***	9.6035	9.3746	1.11
CEO_tenure	5.5144	5.4566	0.65	5.4797	5.4766	0.03	5.4645	5.8533	−1.84*
CEO overconfidence	0.3996	0.4667	−6.52***	0.4672	0.3854	7.58***	0.4381	0.4731	−1.65*
CEO_duality	0.3329	0.3742	−4.03***	0.3715	0.3329	3.80***	0.3602	0.3127	1.67*
Ind_director ratio	0.3733	0.3752	−2.11**	0.3749	0.3733	1.38	0.3746	0.3689	2.16**
Size	21.253	21.4105	−7.95***	21.3994	21.2530	6.89***	21.3569	21.1549	3.38***
Firm_age	7.834	5.2884	20.61***	5.2807	8.078	−22.25***	6.2105	5.1054	3.06***
ROE	0.0623	0.0799	−7.78***	0.0800	0.0607	8.35***	0.0733	0.08069	−1.13
Board size	8.4121	8.2723	4.68***	8.2949	8.3802	−2.83***	8.3098	8.7758	−5.47***
Institutional	5.8640	5.2554	5.16***	5.2478	5.9329	−5.69***	5.4824	5.0744	1.2
Is_chairman_family	0.6528	0.9315	−32.08***	0.9249	0.6358	32.40**	0.8292	0.8054	0.91
Patent application	22.2729	22.6283	−2.30**	22.2991	23.1050	−0.65	22.7627	19.0343	1.72*
Family CEO	0.0020	0.1060	−22.24***	0.0973	0.0160	19.93***	0.0695	0.0125	5.94***
Ultimate ownership	44.0069	51.5031	−24.68***	50.7159	44.2634	20.23***	49.0393	42.6515	10.09***

*Significant at level 1% (0.01), **Significant at level 5% (0.05), ***Significant at level 10% (0.10).

### Descriptive Statistics

Table 3 reports the results of the descriptive statistics. In China, the family firms’ investment ratio in R&D was 0.18% of the total assets. LCFFs comprised 32% of our data set, whereas MCFFs consisted 61%. In our data set, 54% of firms had more voting rights than the cash-flow rights with a 0.5367 mean value.

**TABLE 3:** Descriptive statistics.

Variables	Mean	Std. Dev.	Min	Max	VIF
R&D investment	0.0118	0.05039	0	0.4020	–
Lone controller family firms (LCFFs)	0.3234	0.4671	0	1	1.17
Multi-controller family firms (MCFFs)	0.6158	0.4866	0	1	1.17
With excess voting rights	0.5367	0.4986	0	1	–
Leverag	0.3715	0.2058	0.0460	0.8620	1.65
NOB_Meetings	9.5987	3.6643	4	22	1.17
CEO_tenure	5.4786	3.1184	1	14	1.13
CEO overconfidence	0.4355	0.4958	0	1	1.10
CEO_duality	0.3589	0.4797	0	1	1.10
Ind_director ratio	0.3745	0.0525	0.3333	0.5714	1.39
Size	21.3523	1.0095	18.8105	24.2509	1.56
Firm_age	6.1789	5.8926	1	22	1.88
ROE	0.0735	0.1145	−0.5196	0.4553	1.21
Board size	8.3223	1.4356	5	12	1.45
Institutional	5.4708	5.2023	0.121	26.609	1.14
Is_chairman_family	0.8285	0.4624	0	1	1.26
Patent application	22.5109	40.6577	0	290	1.12
Family CEO	0.0659	0.2482	0	1	1.15
Ultimate ownership	48.6528	15.5436	15.5886	86.1604	1.49

Table 4 shows pairwise correlations of the data set. The correlation analysis demonstrates that our sample data set was free from multi-collinearity, which is a fundamental prerequisite of regression analysis. The correlation analysis of our results discloses statistical correlations among our study variables, control variables, and R&D investment. Moreover, these statistically significant results express the significance of these variables in our regression model. The dependent-variable R&D investment is positively correlated with the LCFFs. The MCFFs were negatively correlated. The moderating variable showed significant results but with excess voting rights negatively correlated, whereas those without excess voting rights were positively correlated.

**TABLE 4:** Correlations.

Variables	(1)	(2)	(3)	(4)	(5)	(6)	(7)	(8)	(9)	(10)	(11)	(12)	(13)	(14)	(15)	(16)	(17)	(18)	(19)
R&D Investment	1																		
LCFFs	0.03*	1																	
MCFFs	−0.05*	−0.87*	1																
With Excess	−0.03*	0.21*	−0.18*	1															
Leverage	−0.05*	0.08*	−0.06*	0.21*	1														
NOB_meetings	0.11*	0.05*	−0.04*	0.02	0.26*	1													
CEO_tenure	0.01	0	−0.01	0.03	−0.01	−0.12*	1												
CEO overconfidence	0.02	0.04*	−0.05*	0.05*	0.06*	0.04*	−0.14*	1											
CEO_duality	0.04*	−0.03*	0.05*	−0.12*	−0.12*	−0.04*	0.09*	−0.10*	1										
Ind_director Ratio	0.05*	−0.01	0.02	−0.08*	−0.04*	0.02	0	−0.04*	0.10*	1									
Size	0.05*	−0.06*	0.08*	0.11*	0.37*	0.32*	0.05*	0.03	−0.08*	−0.06*	1								
Firm Age	0.05*	0.22*	−0.19*	0.29*	0.44*	0.18*	0	0.12*	−0.16*	−0.01	0.19*	1							
ROE	−0.02	−0.08*	0.07*	0.01	−0.10*	0	0.04*	−0.03	0.01	−0.02	0.15*	−0.10*	1						
Board Size	−0.04*	0.02	−0.05*	0.08*	0.09*	−0.02	0.08*	0.04*	−0.11*	−0.59*	0.16*	0.02	0.05*	1					
Institutional	0.06*	0.06*	−0.06*	0.03*	0.02	0.08*	0.07*	0.02	−0.01	−0.01	0.09*	0.11*	0.11*	0.03	1				
Is_Chairman_Family	−0.01	−0.29*	0.28*	−0.19*	−0.14*	−0.06*	0.03	−0.07*	0.10*	0.03*	0.04*	−0.32*	0.09*	−0.02	−0.02	1			
Patent application	0	0	0.01	0.06*	0.10*	0.04*	0.08*	−0.03	0.04*	0.02	0.23*	0.01	0.10*	0.07*	0.01	−0.05*	1		
Family CEOs	−0.04*	−0.18*	0.20*	0.01	0.01	−0.07*	0.05*	−0.03*	−0.11*	−0.02	0.03*	−0.02	0	0.02	−0.03*	0.18*	−0.02	1	
Ultimate ownership	−0.09*	−0.19*	0.23*	0.01	−0.15*	−0.05*	−0.03	−0.08*	0.08*	0.03*	0.08*	−0.40*	0.19*	−0.06*	−0.15*	0.18*	0.04	0.03*	1

*Significance at the 0.1 level.

### Regression Results

To test our hypothesis, we applied the pooled regression model because our study variables were binary (dichotomous). Table 5 shows the regression results of the different types of family firms. The LCFFs show negative significant output. MCFFs also have a significant relationship and act positively with R&D investment.

**TABLE 5:** Regression results without moderation.

	M1	M2
		
Variables	R&D	R&D
Lone controller family firms (LCFFs)	−0.00437*	
	(0.00223)	
Multi-controller family firms (MCFFs)		0.00478**
		(0.00241)
Leverage	−0.03050***	−0.03100***
	(0.00866)	(0.00788)
NOB_meetings	0.00044	0.00056
	(0.00032)	(0.00038)
CEO_tenure	0.00018	0.00002
	(0.00032)	(0.00033)
CEO overconfident	0.00254	0.00222
	(0.00224)	(0.00241)
CEO_duality	−0.00120	−0.00070
	(0.00188)	(0.00226)
Ind_director ratio	0.04020**	0.01720*
	(0.01830)	(0.01980)
Size	0.00407**	0.00364*
	(0.00198)	(0.00185)
Firm age	−0.00036	−0.00018
	(0.00029)	(0.00030)
ROE	−0.01880**	−0.02810***
	(0.00861)	(0.01020)
Board size	−0.00156*	−0.00200*
	(0.00089)	(0.00111)
Institutional	−0.00009	−0.00007
	(0.00022)	(0.00015)
Is_chairman_family	−0.00646**	−0.00574*
	(0.00311)	(0.00307)
Patent application	−0.00001	−0.00001
	(0.00001)	(0.00001)
Family CEOs	−0.00620***	−0.00737***
	(0.00188)	(0.00182)
Ultimate ownership	−0.00018*	−0.00022**
	(0.00009)	(0.00009)
Constant	−0.05730*	−0.04310
	(0.03100)	(0.03090)
Year	Yes	Yes
Industry	Yes	Yes
R-squared	0.135	0.120

Robust standard errors are in parenthesis. ***, **, and * indicate P < 1, 5, and 10%.

In Table 1, for regression output, we used binary (dichotomous) values of our moderating variables. We intended to assess the relationship among the family firms’ types and R&D investments whilst considering the moderating effect of with excess voting and without excess voting rights. We intended to assess the relationship among the family firms’ types and R&D investments whilst considering the moderating effect of with excess voting rights and without excess voting rights with the help of binary (dichotomous) moderating variable. Therefore, in Table 1, we used 1 for with excess voting rights and 0 for without excess voting rights. The LCFFs × with excess voting rights (H1) have negative behaviour to R&D investment in model M3 and support hypothesis H1. The MCFFs × with excess voting rights (H2) show positive behaviour to R&D investment in model M4, and our hypothesis H2 is rejected. By contrast, the LCFFs without excess voting rights have a positive effect on R&D investment. This means that the LCFFs without excess voting rights are more willing to invest in R&D. The MCFFs without excess voting rights have a negative effect. Thus, their willingness is negative toward investment in R&D.

The findings suggest that LCFFs without excess (cash-flow rights) voting rights have the ability and willingness to invest in R&D. When the LCFFs have excess voting rights, then the behaviour regarding R&D investment changes and LCFFs demonstrate less willingness. The LCFFs with excess voting rights invest less in R&D. Based on the results, the MCFFs without excess voting rights have a significant impact and behave negatively toward R&D investment. Their willingness toward R&D investment was lower. According to model M4, MCFFs having excess voting rights act positively regarding R&D investment at a 5% significance level.

They were more willing to invest in R&D with excess voting rights. Consequently, our hypothesis H2 is rejected. However, we indicated earlier in H2 that MCFFs weaken the negative relationship in R&D investment where excess voting rights exist. In the process of weakening the negative relationship, it turned positive in the Chinese MCFFs. Our hypothesis H2 is rejected, which means that Chinese MCFFs want to invest more in risky long-term projects in the presence of excess voting rights. Therefore, our hypothesis H2 negates behavioural agency theory. This finding is our notable contribution to the literature, as detailed in the “Discussion” section, why it happened.

In Table 6, we checked our results for robustness. This time we used continuous values of our moderating (excess voting rights) variables. Our results here are quantitatively similar to the results presented in Table 1. LCFFs × with excess voting rights also have negative behaviour to R&D investment. MCFFs × with excess voting rights show positive behaviour to R&D investment.

**TABLE 6:** Regression results with continuous moderating variables.

	M5	M6	M7	M8
		
Variables	R&D	R&D	R&D	R&D
		
	Main results		Results verified	
		
	With excess voting rights	With excess voting rights	Without excess voting rights	Without excess voting rights
Lone controller family firms (LCFFs)	0.00239		−0.02600***	
	(0.00402)		(0.00673)	
Multi-controller family firms (MCFFs)		−0.00114		0.02610***
		(0.00353)		(0.00678)
Excess Voting Rights (with)	0.00958	−0.01700**		
	(0.00648)	(0.00785)		
Cash Flow Rights (without)			−0.00958	0.01700**
			(0.00648)	(0.00785)
LCFFs × With excess voting rights	−0.02840***			
	(0.00940)			
MCFFs × With excess voting rights		0.02720***		
		(0.00875)		
LCFFs × Without excess voting rights			0.02840***	
			(0.00940)	
MCFFs × Without excess voting rights				−0.02720***
				(0.00875)
Leverage	−0.03140***	−0.03160***	−0.03140***	−0.03160***
	(0.00869)	(0.00866)	(0.00869)	(0.00866)
NOB_meetings	0.00047	0.00046	0.00047	0.00046
	(0.00031)	(0.00031)	(0.00031)	(0.00031)
CEO_tenure	0.00031	0.00032	0.00031	0.00032
	(0.00032)	(0.00032)	(0.00032)	(0.00032)
CEO overconfident	0.00385	0.00390	0.00385	0.00390
	(0.00250)	(0.00253)	(0.00250)	(0.00253)
CEO_duality	−0.00184	−0.00189	−0.00184	−0.00189
	(0.00205)	(0.00205)	(0.00205)	(0.00205)
Ind_director ratio	0.02960	0.02920	0.02960	0.02920
	(0.01890)	(0.01880)	(0.01890)	(0.01880)
Size	0.00390*	0.00392*	0.00390*	0.00392*
	(0.00200)	(0.00200)	(0.00200)	(0.00200)
Firm age	−0.00028	−0.00029	−0.00028	−0.00029
	(0.00033)	(0.00033)	(0.00033)	(0.00033)
ROE	−0.02350**	−0.02340**	−0.02350**	−0.02340**
	(0.00954)	(0.00948)	(0.00954)	(0.00948)
Board size	−0.00223*	−0.00221*	−0.00223*	−0.00221*
	(0.00114)	(0.00114)	(0.00114)	(0.00114)
Institutional	−0.00003	−0.00003	−0.00003	−0.00003
	(0.00022)	(0.00022)	(0.00022)	(0.00022)
Is_chairman_family	−0.00732**	−−0.00735**	−0.00732**	−0.00735**
	(0.00322)	(0.00322)	(0.00322)	(0.00322)
Patent application	0.00001	0.00001	0.00001	0.00001
	(0.00001)	(0.00001)	(0.00001)	(0.00001)
Family CEOs	−0.00715***	−0.00757***	−0.00715***	−0.00757***
	(0.00206)	(0.00211)	(0.00206)	(0.00211)
Ultimate ownership	−0.00015	−0.00015	−0.00015	−0.00015
	(0.00010)	(0.00010)	(0.00010)	(0.00010)
Constant	−0.04650	−0.04510	−0.03690	−0.06210*
	(0.03260)	(0.03250)	(0.03560)	(0.03500)
Year	Yes	Yes	Yes	Yes
Industry	Yes	Yes	Yes	Yes
R-squared	0.133	0.133	0.133	0.133

Robust standard errors are in parenthesis. ***, **, and * indicate P < 1, 5, and 10.

**FIGURE 1:**
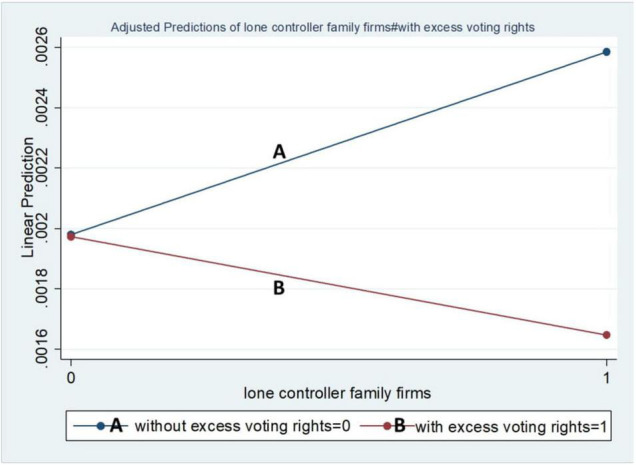
Moderating effect of voting rights on LCFF-R&D investment.

**FIGURE 2:**
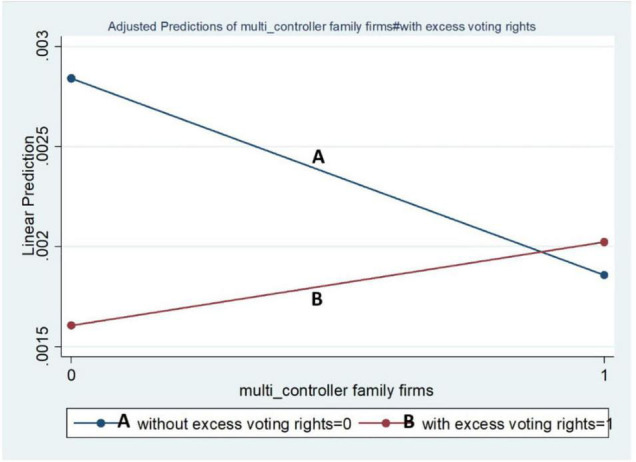
Moderating effect of voting rights on MCFF-R&D investment.

The graphical representations of our results also support the regression results. These graphs were run by dichotomous (binary) moderating variables. Figure 1 represents the LCFFs and the impact of moderating variables, while Figure 2 represents the MCFFs. From Figure 1, LCFFs showed a positive relationship in the absence of excess voting rights and keenness to invest in R&D than the others, whereas the LCFFs with excess voting rights demonstrated negative behaviour regarding R&D. The MCFFs declined in the absence of excess voting rights but were positive in the presence of excess voting rights.

We reran models M3 and M4 by the Tobit regression model for robustness. We censored our dependent variable R&D investment by the upper value. The Tobit model is proposed to estimate the linear relationship among variables when either right or left censoring occurs in the dependent variable. A similar approach was used by Bozec and Di Vito (2018). Table 7 presents the output of the Tobit regression model, and our results in models M9 and M10 remained quantitatively similar to M3 and M4.

**TABLE 7:** Robust regression results with the Tobit model by using dummy moderating variables.

	M9	M10
		
Variables	R&D	R&D
Lone controller family firms (LCFFs)	0.00344	
	(0.00328)	
Multi-controller family firms (MCFFs)		−0.00248
		(0.00309)
With excess voting rights	0.00159	−0.00896***
	(0.00253)	(0.00342)
LCFFs × With excess voting rights	−0.01120***	
	(0.00430)	
MCFFs × With excess voting rights		0.01120***
		(0.00414)
Leverage	−0.03050***	−0.03090***
	(0.00686)	(0.00686)
NOB_meetings	0.00046	0.00045
	(0.00031)	(0.00031)
CEO_tenure	0.00022	0.00023
	(0.00034)	(0.00034)
CEO overconfident	0.00351	0.00361*
	(0.00217)	(0.00217)
CEO_duality	−0.00199	−0.00202
	(0.00224)	(0.00224)
Ind_director ratio	0.02630	0.02760
	(0.02240)	(0.02240)
Size	0.00392**	0.00395**
	(0.00164)	(0.00164)
Firm age	−0.00025	−0.00025
	(0.00033)	(0.00033)
ROE	−0.02160*	−0.02180*
	(0.01290)	(0.01290)
Board size	−0.00224***	−0.00218***
	(0.00069)	(0.00069)
Institutional	−0.00009	−0.00008
	(0.00018)	(0.00018)
Is_chairman_family	−0.00702***	−0.00715***
	(0.00264)	(0.00264)
Patent application	0.00001	0.00001
	(0.00002)	(0.00002)
Family CEO	−0.00660	−0.00702
	(0.00426)	(0.00428)
Ultimate Ownership	−0.00014*	−0.00015**
	(0.00007)	(0.00007)
Constant	−0.04490	−0.04350
	(0.03540)	(0.03530)
Year	Yes	Yes
Industry	Yes	Yes

Robust standard errors are in parenthesis. ***, **, and * indicate P < 1, 5, and 10%.

## Discussion

According to our predicted results, all family-controlled firms use the ability to make investment decisions, but the behaviour of family firms varies in terms of willingness. Our results are quantitatively approved when the LCFFs have voting rights equal to cash-flow rights. Here, firms are more willing to invest in R&D and have positive R&D (Appendix-A). Similarly, we conclude that Chinese multi-controller family firms with excess voting rights also have the ability and willingness to invest in R&D. MCFFs have a positive sign regarding R&D investment (refer to Appendix-A). Schmid et al. (2014) indicated that family-controlled firms pursue a greater level of R&D and that the family firms commit under-reporting because of financial constraints.

The LCFFs have a reduced willingness to invest in R&D when having excess voting rights; the LCFFs have a negative R&D sign (refer to Appendix-A). Similarly, MCFFs without excess voting rights also have less readiness to invest in R&D. MCFFs without excess voting rights have a negative sign (refer to Appendix-A). According to behavioural agency theory, the family firm’s primary concern is to preserve socio-emotional wealth (Block, 2012; Bozec and Di Vito, 2018). In these situations, family firms show less interest in taking risky and long-term decisions. The firms may have several chances to participate in accrual-based earnings management with rewarding returns. In this way, the family firms take a particularistic, family-oriented decision. Consequently, our results conclude that both LCFFs without excess voting rights and multi-controller family firms with excess voting rights have the ability and willingness to engage in R&D investment and invest more significantly. Therefore, LCFFs with excess voting rights and multi-controller family firms without excess voting rights only have the ability, but they have significantly lower willingness toward R&D investment. The previous study investigated that the controllers significantly differ with respect to behavioural shapes such as investment horizons, and risk preferences usually depend on their objectives (Hoskisson et al., 2002).

Few previous studies investigated the basis of agency theory; the lone-founder family firms have a positive relationship with R&D investment (Miller et al., 2011; Block, 2012). Moreover, some other studies based on agency theory suggest that family-controlled firms have a negative relationship with R&D investment (Muñoz-Bullón and Sanchez-Bueno, 2011; Choi et al., 2015; Broekaert et al., 2016). In these studies, the scholars assumed that both types of family-controlled firms have both ability and willingness conditions, but these studies did not separately explore the willingness condition. Hence, agency theory concentrates on the sole detection of economic goals. Consequently, agency theory proposes that when the ability condition is met, then the willingness condition must be present. While examining the family firm’s behaviour and ignoring the willingness condition, this process might be the critical limitation of agency theory (Bozec and Di Vito, 2018). By adding this contribution to the existing literature, we consequently challenge this implicit assumption, and our results prove that the willingness to invest in R&D varies between different kinds of family businesses in the absence or existence of excess voting rights. Specifically, when LCFFs have no excess voting rights and MCFFs have excess voting rights, the ability and willingness conditions are satisfied, and the firms invest more in R&D.

Thus, distinguishing among LCFFs and MCFFs is essential in the presence and absence of excess voting rights to recognise when both ability and willingness conditions exist. With an increase in excess voting rights, agency cost and family firms are empowered to make a decision, which is most favourable to their families and personal wealth, while the cost of investment decisions might be externalised to the firm as a whole. The picture is clear about the LCFFs without excess voting rights; when the controllers have no other family members in the firm, then the controllers make decisions individually. These decisions might be risky long-term decisions, and they have both the ability and willingness to invest in R&D. The other reasons are that when the LCFFs have no excess voting rights, these firms are normally younger firms and invest more in R&D investment to attract the shareholders’ attention, new product development, and market share. More importantly, because of these factors, controllers attempt to obtain more control rights (Czarnitzki and Hottenrott, 2011). The study finds that the new entrants probably pursue other innovative and even more beneficial goals for competitiveness (Higón, 2012). Aziz and Samad (2016) argued that the new entrants behave belligerent, proactive, and flexible.

In contrast, MCFFs also have both the ability and willingness to invest in R&D when they have excess voting rights. When the MCFFs have excess voting rights, they have more ownership concentration. As already discussed, ownership concentration is a powerful tool for particularistic family decisions (Min, 2021). In this situation, agency problems typically arise because many family members from different positions are involved in the decision-making process. However, our hypothesis (H2) was rejected, thereby indicating that when Chinese MCFFs have excess voting rights, they take decisions beyond the agency problems. The MCFFs tend to make decisions for the benefit of their families and minority shareholders. According to Chen et al. (2013), family firms with positive voting-cash flow rights divergence engage in innovative investment projects. The authors further found that innovation behaviour is observed in Taiwanese family firms more than in US family firms in terms of long-run presence concern and lower-risk diversification.

The following describes our notable contributions to the existing literature. When the family firms are caught in challenging situations such as (1) a smaller earning failure (decline) or loss and (2) a debt contract violation, in Taiwan firms, the family firm’s owner decreases myopic behaviour in R&D investment (Tsao et al., 2019). The decline in the smaller earnings may increase the risk of take over due to undervaluing of stock (Stein, 1988). The family-controlled firms already have significant incentives to save the family firm and the family’s reputation and also to evade the activities that decrease the long-term firm value; moreover, this study also evidenced that when the family firms have limited opportunities to be involved in accrual-based earnings management, they continue to evade myopic R&D reduction (Tsao et al., 2019). The high institutional ownership often acts as a monitor, and the managers are less likely to reduce the R&D spending to reverse the earnings decline (Bushee, 1998). The shortfall in performance usually motivates family firms to invest in R&D (Gomez–Mejia et al., 2014). Ownership is regarded to have the potential to influence the future path of corporate operations, which might have an impact on the company’s financial and innovation strategy (Tahir and Sabir, 2015).

Another critical concern is that LCFFs with excess voting rights (H1) are less willing to build R&D investments. Could the presence of excess voting rights in LCFFs hinder the willingness to invest in R&D? The possible reason for these outcomes is that LCFFs with excess voting rights are usually older than LCFFs without excess voting rights. Furthermore, LCFFs also attempt to attain more control rights. Once LCFFs gain the latter, then the R&D investment behaviour of these firms also decreases. Firm age and innovation output are inversely related (Hansen, 1992). When a firm grows, shares are traded publicly, and the controller of the firm may want to retain control. In these situations, the lone controller uses control-enhancing instruments such as dual-class shares, which may be necessary to warrant their holding control of the firm. The extra empowerment or intense ownership concentration extended to the excess voting rights permits the lone controller to externalise the cost of the suboptimal but self-gainful investment. No other additional concern exists regarding adjusting to family-controlled wealth. Di Vito et al. (2010) stated that the level of difference between voting rights and cash-flow rights for controlling shareholders should have an inverse relationship with R&D investment decisions. They further justified that the difference between voting rights and cash-flow rights provides controlling shareholders an extra advantage in the form of power and incentive, which they exercise to obtain extra benefits at the cost of minority shareholders.

## Conclusion

We summarise the conclusion of our study using (De Massis et al., 2014) family firms’ particularistic behaviour model. Moreover, we applied this model to identify the behaviour of different types of family firms regarding R&D investment. In our study, to increase the higher value of firms, family firms should keep investing in R&D. We examined the R&D investment behaviour of lone and multi-controller family firms with and without excess voting rights. We also investigated whether different types of family firms with and without excess voting rights have both the ability and willingness to invest in R&D. According to the above-discussed model, with the presence of both sufficiency conditions (ability and willingness), all types of family firms can invest in R&D but differ in terms of willingness. Our results indicate that the LCFFs without excess voting rights and multi-controller family firms with excess voting rights have both the ability and willingness. Thus, these two firms are more willing to invest in R&D. By contrast, LCFFs with excess voting rights and multi-controller family firms without excess voting rights only have the ability. These firms are less willing to invest in R&D. Family firms in China are heterogeneous regarding their investment behaviour in R&D, both with and without excess voting rights.

Our findings also indicate that when the LCFFs are endowed with excess voting rights, they change their willingness conditions and show less interest in investing more in risky long-term projects. Multi-controller family firms with excess voting rights also change their willingness to R&D investment. Interestingly and surprisingly, multi-controller family firms’ behaviour toward R&D investment changes to positive. Multi-controller family firms have significant incentives to save both the family firm and the family’s reputation and also to evade activities that decrease the long-term firm value.

Our quantitative results add significant contributions to the existing literature. This study presents an overall direction for further understanding by examining the family firms’ behaviour regarding R&D investment in enhancing firm value. To the best of our knowledge, this study is unique in Chinese family firms’ behaviour in the presence and absence of excess voting rights regarding R&D investment by using the family firms’ particularistic behaviour model described by De Massis et al. (2014). Our results can be generalised, especially in Asia, where the family firms have characteristic features of ownership structures and also have excess voting rights. We collected data from 2008 to 2018, which implies that our results are recent and relevant to the current economic situation.

Our study has practical implications. This study has important implications for firm owners/controllers because it demonstrates that controlling rights are crucial for determining the R&D investment behaviour of LCFF and MCFF. Our findings suggest that family control firms engage in protecting their socio-emotional wealth and decreasing their willingness to invest in R&D. An implicit assumption exists that a combination of control and ownership rights empowers the family firms with legal rights over the firms’ resources and profits (Carney, 2005). This phenomenon results in control over the willingness of family firms to invest in R&D. Nevertheless, for the Chinese MCFF, our hypothesis H2 contradicts the behavioural agency theory when these firms suffer under unusual conditions, even in the presence of excess voting rights. Family firms are predominant worldwide, and several countries’ economic growth depends on family-controlled firms. This study also motivates future researchers to categorise family firms based on different characteristics in order to measure the varying behaviour in different types of family firms. Our study will be beneficial to future investors, who can easily predict the value and benefit of investments. This study will help investors to know which types of family firms are willing to invest in R&D. Innovation in family firms plays a vital role in economic growth. The only way for family firms to enhance their firms’ value is by investing in R&D.

## Limitations

This study has some limitations that provide future research opportunities. First, this study has focused on Chinese family firms only. Second, family firms can be categorised based on different characteristics, whereas in this study, family firm types have been considered based on controlling rights only. With respect to controlling rights, family firms are categorised into three categories. However, the researcher has considered only two types due to the study objective and requirements. The data have been collected till 2018 due to the non-availability of data for the latest years. Moreover, this study has produced quantitative results, which is also the limitation of this study as it has not dealt with qualitative results, which is suggested for future researchers.

## Data Availability Statement

The original contributions presented in this study are included in the article/supplementary material, further inquiries can be directed to the corresponding author.

## Author Contributions

All authors listed have made a substantial, direct, and intellectual contribution to the work, and approved it for publication.

## Conflict of Interest

The authors declare that the research was conducted in the absence of any commercial or financial relationships that could be construed as a potential conflict of interest.

## Publisher’s Note

All claims expressed in this article are solely those of the authors and do not necessarily represent those of their affiliated organizations, or those of the publisher, the editors and the reviewers. Any product that may be evaluated in this article, or claim that may be made by its manufacturer, is not guaranteed or endorsed by the publisher.

## Appendices

**Appendix A:** Predicted behaviour of different types of family-controlled firms based on the sufficiency condition model described by De Massis et al. (2014).

Family Firms Types	Ability	Willingness	R&D Sign
Lone-controller family firms without excess voting rights	Yes	Yes	+
Multi-controller family firms without excess voting rights	Yes	No	−
Lone-controller family firms with excess voting rights	Yes	No	−
Multi-controller family firms with excess voting rights	Yes	Yes	+
			

**Appendix B:** Definition of variables.

Terms	Definition
R&D investment	Total R&D expenditures divided by total assets at current year- end.
Lone-controller family firms	Family-owned firms whose actual controller is an individual except controlling other management positions held by family members.
Multi-controller family firms	Multi-family members are the actual controllers, and a minimum of one family member with kinship holds, manages, and controls the family business of the listed company or the supervisory shareholder company.
Other-type family firms	The actual controller is a plurality of natural persons, but the actual controller has no kinship relationship.
Without excess voting rights	A case where the family firm’s controllers have voting rights equal to the cash flow rights.
With excess voting rights	A case where the family firm’s controllers have voting rights more than the cash flow rights.
Ownership proportion	The proportion of cash flow rights held by the family firm’s controllers at the end of the current year.
Controlling proportion	The proportion of voting rights held by the family firm’s controllers at the end of the current year.
Separation proportion	Family firms have voting rights higher than cash flow/ownership rights. In this situation, the firm is supposed to be endowed with the existence of excess voting rights.
Leverag	The total debt divided by total assets.
NOB_meetings	The total number of board meetings in a given year.
CEO_tenure	The number of years that the current CEO held his or her position at the end of his or her tenure.
CEO_duality	If the CEO is also the chairman of the firm.
Ind_director ratio	The independent director ratio measured as board size scaled by the number of independent director ratio.
Size	The log of total assets at the end of the previous year.
Firm_age	The number of years the firm has been in operation.
ROE	The net earnings divided by equity at the previous year-end.
Board size	The total board size of the firm in the current year.
Institutional	The total proportion of voting shares held by institutional investors at the end of the current year.
Is_chairman_family	If the chairman of the firm is a family member or not.

**Appendix C:**

The actual controller has a percentage of ownership of the listed company (%) known as cash-flow rights. It refers to the ownership of a scheduled company owned by the actual controller through concerted action, multiple holdings and cross-shareholdings. The values of cash-flow rights have been directly taken from CSMAR. The CSMAR database reflects the calculation of the cash-flow proportion of the family-controlled firms based on calculation methods (Claessens et al., 1999, 2000; La Porta et al., 1999). This study showed the examples below by Aslan and Kumar (2012) also calculated voting rights by following (Claessens et al., 1999, 2000; La Porta et al., 1999).
**Example 1: Pyramidal-holding** Firm X Firm Y (40%) Firm Y Firm Z (15%) Firm X’s cash-flow (CF) and control (C) rights in Firm Z CF (shares owned): 6% (=40%*15%) C (votes controlled): 15% (=min(15%,40%)) Deviation of cash-flow from control rights: 15%/6% = 2.5
**Example 2: Cross-holding** Firm X Firm W (40%) Firm W Firm Z (15%) Firm X Firm Y (20%) Firm Y Firm Z (10%) Firm X’scash-flow (CF) and control (C) rights in Firm Z CF (shares owned): 8% ((=(20%,10%)+(40%,15%)) C (votes controlled): 25% (=min(20%,10%)+min(40%,15%)) Deviation of cash-flow from control rights: 25%/8% = 3.1
**Example 3: Circular-holding** Firm X Firm Y 35% Firm Y Firm 10% Firm Z Firm X 8% Firm X’s cash-flow (CF) and control (C) rights in Firm Z CF (shares owned): 3.5% ((=(35%*10%)) C (votes controlled): 25% ((=min(35%,10%)) Deviation of cash-flow from control rights: 25%/3.5% = 7.14 In turn, Z also has 8% of cash-flow and control rights in X.
**Example 4: Dual-class holdings** Firm X Firm Y 60% Firm Y Firm Z Ownership: 30 % Voting: 80% Firm X’s cash-Flow (CF) and control (C) rights in Firm Z CF (shares owned): 18% ((=(60%*30%)) C (votes controlled): 60% ((=min(80%,60%)) Deviation of cash-flow from control rights: 60%/18% = 3.33
